# The Muscular Retractive Power in the Bowels Considered in Its Relation to Strangulated Hernia

**Published:** 1884-09

**Authors:** Frank H. Nichols

**Affiliations:** Atlanta, Ga.


### THE MUSCULAR RETRACTIVE POWER IN THE BOWELS CONSIDERED IN ITS RELATION TO SRANGULATED HERNIA.

BY FRANK H. NICHOLS, M. D., ATLANTA, GA.

We wish to place before the readers of the ATLANTA MEDICAL AND SURGICAL JOURNAL some of the results obtained by a careful research and study of the muscular movements of the bowels. We believe these results will be of benefit to some, perhaps of interest and amusement to others. They were made under the most carefully considered circumstances, with a single purpose to ameliorate human suffering and save human life.

The first result established was that the normal action or movement of the bowels is forward, and a backward movement the result of muscular contraction. The second result was found to be a product of these two movements—a blending of these two-in what is described "a vermicular motion." This vermicular movement becomes the truly normal movement of the bowels, in consequence of their hinge-like attachment within the abdomen.

The third result established was that all normal movement carried the contents of the bowels onward.

The fourth result obtained was that the bowels possessed a retractive, or pull-back power within the abdomen. Here our experiments ended.

Accepting the above statements as true, we have passed over into the domain of speculative philosophy and wrought out some wonderful results in medical practice. The first result worked out was to show that the natural retractive power of the bowel was salutary. This was made to appear in a severe case of strangulated hernia. Taxis, as taught in medical books, was carefully and repeatedly made. The hernia was not reduced The patient was apparently doomed to a surgical operation or death. The operation was rejected by the patient who preferred to die as he was. This led to another trial, combining the retractive power of the bowels with taxis—reduction followed with relief and recovery. This result has now been worked out with so many other cases that we can safely announce this law as established: "*The retractive power of the bowels, aided by proper taxis, will certainly reduce all cases of strangulated hernia.*" This corollary follows: The normal retractive muscular force exerted by the bowels in hernia is conservative of human life.

The next result shown was the absolute obedience and control of this retractive power in strangulated hernia. It is a matter of demonstration at the bed-side. They all recover.

We can but admire that Infinite Wisdom and goodness deep planted in man's organism by the Creator, who in the beginning so adjusted the power of muscular contraction as to best serve and promote, by active industry, individual health and happiness—and still so balanced this power that it truly becomes eonservative amid the accidents and misfortunes of this life.

